# Disrupted autophagy undermines skeletal muscle adaptation and integrity

**DOI:** 10.1007/s00335-016-9659-2

**Published:** 2016-08-02

**Authors:** Elliot J. Jokl, Gonzalo Blanco

**Affiliations:** Department of Biology, University of York, Wentworth Way, York, YO10 5DD UK

## Abstract

This review assesses the importance of proteostasis in skeletal muscle maintenance with a specific emphasis on autophagy. Skeletal muscle appears to be particularly vulnerable to genetic defects in basal and induced autophagy, indicating that autophagy is co-substantial to skeletal muscle maintenance and adaptation. We discuss emerging evidence that tension-induced protein unfolding may act as a direct link between mechanical stress and autophagic pathways. Mechanistic links between protein damage, autophagy and muscle hypertrophy, which is also induced by mechanical stress, are still poorly understood. However, some mouse models of muscle disease show ameliorated symptoms upon effective targeting of basal autophagy. These findings highlight the importance of autophagy as therapeutic target and suggest that elucidating connections between protein unfolding and mTOR-dependent or mTOR-independent hypertrophic responses is likely to reveal specific therapeutic windows for the treatment of muscle wasting disorders.

**Electronic supplementary material:**

The online version of this article (doi:10.1007/s00335-016-9659-2) contains supplementary material, which is available to authorized users.

## Introduction

Skeletal muscle functions as both an essential force-generating tissue and the body’s primary protein reservoir. As such, it has evolved the plasticity necessary to adapt itself according to the mechanical and metabolic demands placed upon it, within the context of the physiological needs of the whole organism.

Mechanosensitive adaptations in skeletal muscle are broadly determined by the relative balance between hypertrophic mTOR signalling and pro-degradation AMPK signalling. The mTOR pathway is activated by mechanical tension (Baar et al. 1999, 2000). The activation of mTOR leads to an increase of protein synthesis for up to 72 h above rest (Miller et al. 2005) and this positive net balance leads to muscle fibre hypertrophy of mainly type 2 fibres. In contrast, inhibition of mTOR with rapamycin prevents an increase in protein synthesis and compensatory hypertrophy in rodents and humans (Bodine et al. 2001; Drummond et al. 2009). Activation of mTOR hence appears to be a necessary contributing factor (e.g. Frey et al. 2014) for load-induced muscle growth, but the actual mechanosensor or mechanotransduction mechanisms remain elusive. Conversely, when muscle is unused, AMPK inhibits the mTOR pathway. This results in the upregulation of the two major proteolytic pathways, autophagy and the Ubiquitin Proteasome System (UPS), via the FOXO transcription factors and leads to muscle atrophy. These proteolytic pathways are also upregulated in response to low availability of amino acids and other nutrients (Milan et al. 2015; Sandri et al. 2004; Stitt et al. 2004).

Functional degradative pathways are required for atrophy and muscle maintenance. Muscle protein synthesis also relies on the availability of free amino acids, which are at least in part obtained via the breakdown of old and damaged proteins and organelles (see Schiaffino et al. 2013 for a review). Atrophy relies on degradative pathways as primary effectors. In addition, muscle maintenance relies on a baseline turnover of mechanically unfolded proteins to prevent cytotoxic accumulation of aggregates, as well as the turnover of damaged organelles, particularly mitochondria.

In this review, we discuss the importance of autophagy in skeletal muscle with a particular emphasis on the genetic evidence showing the sensitivity of muscle tissue to mutations that disrupt basal and induced autophagy pathways. We also discuss emerging evidence of a form of tension-induced autophagy that links degradation of unfolded protein with the synthesis of its replacement. Given damage to proteins and organelles correlates to muscle usage, it is possible that the relative activation of specific autophagy pathways itself acts as one of the mechanosensing mechanisms required for muscle hypertrophy.

## Review

### Protein turnover in muscle disease

The two major proteolytic systems in skeletal muscle are the autophagosome/lysosome and the UPS. The importance of protein turnover in muscle maintenance can be assessed by quantifying how well represented proteostasis regulation, defined as factors involved in autophagy and the proteasome, is amongst genetic diseases in muscle. This could be gathered by a straightforward bioinformatics analysis of GO terms and cellular components that are overrepresented in the current list of genes underlying neuromuscular disorders (Kaplan and Hamroun 2014). An updated list of muscle disease genes (Kaplan and Hamroun 2014), extended with genes known to cause muscle pathology when targeted in mice but so far lacking an associated human disease (from MGI, http://www.informatics.jax.org/), was submitted to the software package *GOrilla* (Eden et al. 2009) using the reviewed *Homo sapiens* UniProt protein list as reference (http://www.uniprot.org/uniprot).

As expected, many categories of specific cellular processes and cellular components are enriched in the disease list at *p* values below the threshold of significance. Autophagy and the autophagosome emerged as significantly enriched GO term categories for cellular processes and components, respectively, whilst the UPS did not [see Table 1; full input list and output lists of enriched GO terms for cellular processes and components with the corrected *p* values for multiple testing (Benjamini and Hochberg 1995) are shown as supplementary material]. This suggests that muscle maintenance mechanisms are more susceptible to pathogenic mutations in autophagy than in the UPS. This evaluation appears likely to under-report the significance of autophagy in skeletal muscle, as many of the disease genes shown to cause autophagy disruption described below did not emerge in the GO term analysis, e.g. BAG3, MTM1 and VMA21. It is clear that for many disease genes the corresponding GO terms do not fully capture their known functions.

**Table 1:** Selected GO term categories overrepresented in muscle disease genes

Description	Category	GO term	*p* value	FDR *q*-value	Contributing genes
Z-disc	Component	GO:0030018	8.26E-41	6.67E-38	CAPN3, KCNA5, CSRP3, ACTN2, CRYAB, JUP, MYH6, MYH7, CASQ2, MYOZ2, BAG3, FLNC, PSEN2, SMN1, SCN5A, TCAP, CACNA1C, MURC, SCN8A, LDB3, RYR2, BIN1, SCN3B, CAV3, JPH2, TTN, NEB, DMD, KCNE1, DES, HSPB1, SYNE2, NEXN, MYOT, DNAJB6, ANK2, MYPN
Autophagosome	Component	GO:0005776	6.26E-05	1.10E-03	PIK3R4, PIK3C3, ORAI1, C9orf72, OPTN, UBQLN2
Autophagy	Process	GO:0006914	5.62E-04	1.77E-02	PIK3R4, MFN2, PIK3C3, ATG4C, C9orf72, OPTN, UBQLN2, EPG5

FDR *q*-value is the false discovery rate correction of the above *p* value for multiple testing using the Benjamini and Hochberg method (Stitt et al. 2004)

### Autophagy in skeletal muscle maintenance

Three major autophagic pathways are distinguished according to how cargo enters the lysosomes: chaperone-mediated autophagy (CMA), microautophagy and macroautophagy. In CMA, proteins with the KFERQ pentapeptide motif in their sequence, typically exposed by unfolding or denaturing, are recognized by molecular chaperones and directly translocated into lysosomes through the *LAMP2A* (Lysosomal-associated membrane-2 protein) receptor on the lysosomal membrane (Dice 1990). In microautophagy, cytoplasmic components are directly engulfed into the lysosomal lumen. And in macroautophagy, a purpose-built double membrane structure surrounds the cytoplasmic components to form the autophagosome (Mizushima et al. 2008). Autophagosomes then fuse with the lysosome, and the membrane and content of the autophagosome vesicle are degraded. Macroautophagy, commonly and hereafter referred to as autophagy, is induced by starvation and functions to supply amino acids and energy from the bulk degradation and recycling of intracellular components (Klionsky 2007).

It would be expected that autophagy plays a major role in skeletal muscle, as this constitutes the major reservoir of protein and energy in the organism. Indeed, fast-twitching muscles in particular have been shown to be extremely responsive to starvation-induced autophagy in comparison to other tissues, including brain (Mizushima et al. 2004). Moreover, several studies indicate that *basal* autophagy levels vary amongst the different muscle types, likely reflecting their specific physiological demands. Basal autophagy continuously clears out misfolded proteins, protein aggregates and worn-out organelles such as mitochondria during non-starvation conditions. Muscles experiencing continuous tension may therefore be expected to have a higher level of basal autophagy as an adaptation to the presumptive increase in protein unfolding and metabolic strain. Indeed, it has been shown that the expression of autophagy (LC3-I/LC3-II, Beclin-1 and Atg7) and mitophagy (BCL-2/adenovirus E1B–interacting protein-3, abbreviated as Bnip3) proteins is significantly higher in tonic, oxidative muscle (soleus) when compared to a muscle of mixed fibre types (plantaris) or a phasic, glycolytic muscle (vastus lateralis) (Lira et al. 2013). This study concluded that oxidative muscles have a higher autophagic flux, with increased LC3-II/LC3-I ratios and lack of p62 (also known as SQSTM1) accumulation, indicating elevated autophagosome turnover. The data underlying this conclusion are supported by the relative levels of LC3-II and p62 protein and mRNA in oxidative and glycolytic muscles of the control group in a different study (Mofarrahi et al. 2013) and the reports agree that highly oxidative muscles have higher levels of Bnip3 and other mitophagy proteins.

A criticism of (Lira et al. 2013) is that their study represents an indirect way of estimating autophagic flux, generating interpretations from a snapshot of the relative levels of autophagy markers. It has been argued that flux is better measured directly by examining tissue response to autophagy inhibitors, e.g. colchicine (Mofarrahi et al. 2013) and such approaches are considered more robust in published guidelines for interpreting autophagy assays (Klionsky et al. 2016).The effect of such inhibitors is to prevent the degradation of LC3-II, and measuring the resulting accumulation thus provides a dynamic measure of LC3-II synthesis rates. A study using this approach concluded that glycolytic muscles have higher autophagic flux (Mofarrahi et al. 2013), contradicting the interpretations in (Lira et al. 2013). However, this approach may not be suitable in the context of examining the relative baseline autophagy levels between muscle types given that blocking autophagy itself is likely to induce aberrant signalling responses in muscle tissues, particularly with prolonged colchicine treatments. A recent study in macrophages has proposed that colchicine functions to activate AMPK by promoting phosphorylation of LKB1 (Wang et al. 2016). Since fast-twitch muscles are more sensitive to the induction of autophagy, possibly due to higher levels of Ulk1 protein allowing rapid activation of LC3-II biosynthesis (Mizushima et al. 2004; Mofarrahi et al. 2013), the use of colchicine is likely to overestimate genuine basal flux in fast-twitch muscles. Shorter treatments with leupeptin are used to provide similar flux measurements. These are likely to be more robust than those obtained using colchicine, but the question of how well tissues with varying sensitivities to autophagy induction can be compared via this method remains unclear. Until this bias can be controlled for, estimation of flux using snapshots of autophagy markers may represent the more reliable way of comparing basal autophagy between muscle types.

Beyond basal- and starvation-induced autophagy, the accumulation of misfolded proteins that results from cytoskeletal stress induces a specific form of autophagy known as Chaperone-Assisted Selective Autophagy (CASA). As discussed later, CASA has been shown to be particularly relevant for tension bearing cells where it targets specific proteins for degradation such as filamin C (FLNC) (Arndt et al. 2010; Ulbricht et al. 2013). Disruption of autophagy in LAMP-2 knockout mice leads to progressive muscle weakness but, intriguingly, accumulation of aggregated FLNC as well as accumulation of autophagic vacuoles have been reported only in soleus muscle (Arndt et al. 2010; Tanaka et al. 2000). This may indicate that the efficient turnover of structural proteins is more critical in tonically active muscle.

### Disruption of autophagy induces skeletal muscle pathology

**Fig. 1:**
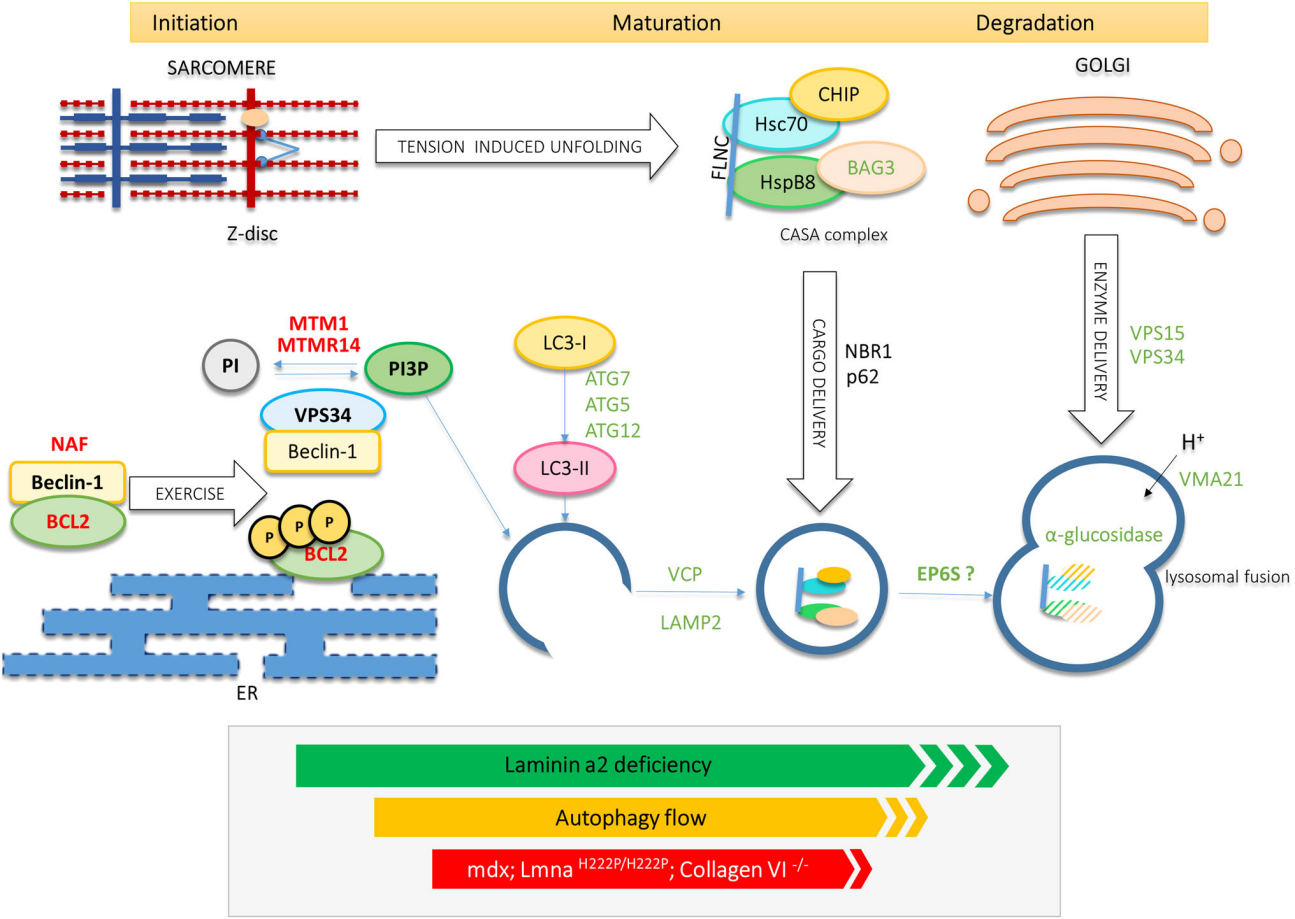
Proteins with known or inferred roles in autophagy that underlie muscle pathologies in mice and humans. Virtually all stages of autophagy, including exercise-induction via phosphorylation of Bcl2, response to sarcomeric protein unfolding via the CASA pathway as well as autophagosome biosynthesis and maturation are targets in muscle disease. Positive regulators and components of the autophagy pathway that are associated with muscle pathology are shown as protein symbols in *green*. Negative regulators which are associated with muscle pathology, as protein symbols in *red*. In addition, the downregulation/impairment of autophagy observed in laminin α2 deficiency, mdx and Lmna^H222P/H222P^ mouse models and upregulation of autophagy in collagen VI deficiency are indicated

Genetic defects that disrupt each of the phases that autophagy encompasses (initiation, maturation and degradation of the lysosomal autophagic content) underlie skeletal muscle disease in mice and humans (Fig. 1). Moreover, the severity of the phenotype may depend on whether the mutation alters basal or an inducible form of autophagy.

In mice, activation of autophagy can be detected just 15 min after acute exercise (He et al. 2012). This rapid activation is underpinned by the disruption of the BCL2–Beclin-1 complex in the endoplasmic reticulum (ER) (Pattingre et al. 2005). Beclin-1 is involved in autophagic vesicle nucleation through its interaction with Vps34 (Kihara et al. 2001), a Class III phosphatidylinositol 3-kinase (PI3K) that converts phosphatidylinositol (PI) to phosphatidylinositol 3-phosphate (PI3P). BCL2 inhibits both the interaction between Beclin-1 and Vps34 and the Beclin-1-associated Vps34 kinase activity (Pattingre et al. 2005). This inhibitory role of BCL2 is released upon the phosphorylation of three key residues within the non-structured loop of the protein (Wei et al. 2008). Mutations in the BCL2 phosphorylation sites (Thr69Ala, Ser70Ala and Ser84Ala) prevented exercise-induced disruption of the BCL2–beclin-1 complex and subsequent activation of autophagy (He et al. 2012). The BCL2 mutations, referred to as BCL2 AAA, did not disrupt basal autophagy; yet, homozygous BCL2 AAA mice not only showed impaired exercise endurance, but also impaired glucose metabolism following a regime of chronic exercise and high-fat diet (He et al. 2012). Maximal running distance in a single session was also significantly lower in BCL2 AAA mice.

However, other methods of autophagy impairment that specifically target skeletal muscle have not confirmed that autophagy is required for an acute bout of exercise or significant changes in glucose homeostasis. These studies used either Beclin-1 heterozygous mice, constitutive muscle specific muscle-specific deletion of Atg7 or inducible muscle-specific deletion of Atg7 (Lira et al. 2013; Kim et al. 2013; Lo Verso et al. 2014). Atg7 is the E1-like enzyme of the ubiquitin-like conjugation systems that activates the E3-like ubiquitin ligase Atg5 to form a complex with Atg12, which is essential for LC3-1 conjugation to phosphatidylethanolamine to form LC3-II (Tanida et al. 2001). Discrepancies between these experiments may rest on the involvement of other organs in the case of the constitutive BCL2 AAA mice, the specific gene being targeted or even the length and timing of autophagy impairment. Indeed, constitutive deletion of Atg7 in skeletal muscle leads to compensatory upregulation of the Fg21 mitokine triggered by accumulation of damaged mitochondria. In turn, Fg21 upregulation promotes effective protection from high-fat diet-induced obesity and insulin resistance (Kim et al. 2013), phenotypic outcomes opposed to those observed in BCL2 AAA mice.

Accumulation of damaged mitochondria is a consistently reported cellular event in autophagy-impaired muscle. In normal muscle, endurance exercise training induces expression of mitochondrial biogenesis markers (evidenced by upregulation of CyC, Cox4 and Pgc1-a) and mitophagy (Bnip3) in mixed fibre type plantaris muscle but not in oxidative soleus muscle (Lira et al. 2013). Autophagy-impaired Beclin-1 ± mice, which are haploinsufficient for the Beclin-1 component of PI3-Kinase complexes involved in autophagy and protein sorting (Cao and Klionsky 2007), do not induce upregulation of those mitochondrial turnover markers. These mice failed to improve endurance capacity when assessed by maximal treadmill running distance (Lira et al. 2013). Similarly, dysfunctional mitochondria accumulated in exercised mice in which Atg7 deletion was induced prior to exercise (Lo Verso et al. 2014). In the latter, training consisted of consecutive bouts of downhill running to induce damaging eccentric contraction, which resulted in decrease in performance in autophagy-impaired mice. Morphologically abnormal, functionally deficient swollen mitochondria have also been shown to accumulate in mice with muscle-specific constitutive deletion of Atg7 (Kim et al. 2013; Masiero et al. 2009; Wu et al. 2009). Thus, a view has emerged that a main role for exercise-induced autophagy in skeletal muscle is to remove damaged mitochondria that would otherwise accumulate and prevent improvement of endurance capacity.

Constitutive impairment of basal autophagy has clear detrimental effects on skeletal muscle although akin to the examples above, differences in phenotypic outcomes have been reported depending on the targeting strategy. For instance, deletion of Atg7 in skeletal muscle driven from the creatine kinase promotor or the myosin light chain fast 1 promotor results in degenerative changes from 40 days (Wu et al. 2009) or longer than one year (Masiero et al. 2009), respectively. The fact that constitutive autophagy is essential to preserve muscle integrity was also demonstrated by the phenotypes of Atg5 KO mice, characterized by muscle loss, protein aggregates and accumulation of numerous aberrant membranous structures (Kihara et al. 2001). An example of detrimental effects caused by excessive autophagy is provided by NAF-1, a small endoplasmic reticulum (ER) transmembrane protein and cofactor required for BCL2 to antagonize Beclin-dependent autophagy at the ER. Lack of NAF-1 triggered an excess of autophagy in non-stimulated conditions. Skeletal muscle, particularly the diaphragm, was amongst the first tissues to show degeneration (Chang et al. 2012).

Defects in later stages of autophagy also result in major muscle pathology. The mammalian orthologue of Vps15 was recently knocked out in mice (Nemazanyy et al. 2013). In yeasts, the Vps15/Vps34 complex is involved in the delivery of soluble hydrolases from the Golgi to the vacuole and is required for endosomal sorting and autophagy (Schu et al. 1993). Vps15 is a phosphoinositide-3-kinase adaptor protein that regulates the activity of Vps34. Though lethal when deleted ubiquitously, muscle-specific deletion of Vps15 resulted in defects in late endosomal/lysosomal functions and accumulation of ultrastructural features reminiscent of lysosomal storage diseases and autophagic vacuolar myopathy (Nemazanyy et al. 2013).

### Autophagy and skeletal muscle disease

In humans, mutations have been identified affecting later steps in the autophagic process. X-linked myopathy with excessive autophagy is caused by haploinsufficiency mutations in the *VMA21* gene, which codes for the transmembrane subunit of the V-ATPase lysosomal proton channel (Ramachandran et al. 2009). The reduced activity of the V-ATPase results in reduced proton influx and an increase of 0.5 units of the lysosomal pH. This higher pH caused impaired degradative power of the lysosome and lower levels of free AAs. The limited AA availability induced autophagy as a compensatory mechanism through mTOR, which leads to excessive autophagy. Given that the pathological findings were restricted to muscle, it was suggested that skeletal muscle is particularly sensitive to the upregulation of autophagy. Muscle susceptibility to autophagic perturbations has indeed been reported in other inherited conditions. Danon disease is characterized by the presence of autophagic vacuoles with sarcolemmal features (Nishino et al. 2000). It is caused by mutations in the major component of the lysosomal membrane *LAMP2*. Although a multisystemic disorder, skeletal muscle and heart are the most affected tissues in patients (Sugie et al. 2002). In mice, lack of this structural lysosomal protein also causes increased postnatal lethality, although some mice survive and have a normal life span. Mice that survive show accumulation of autophagosomes in many tissues, but the prominent pathological manifestations are also found in heart and skeletal muscle (Tanaka et al. 2000). It appears clear that *LAMP2* is required for phagosome and autophagosome fusion, but not for proteolytic function of the lysosome (Eskelinen et al. 2002, 2004).

However, the degradative fitness of the lysosome is also a target in muscle disease. In Pompe disease, the underlying defect is the lysosomal enzyme α-glucosidase (or acid maltase), which hydrolyzes glycogen and maltose to glucose. Pompe described this disease in 1932 when he observed abnormal accumulation of glycogen in all tissues examined from an infant that died from idiopathic hypertrophic cardiomyopathy. Pompe disease affects multiple tissues, but skeletal and cardiac muscles are particularly vulnerable to the accumulation of storage material and the perturbation of autophagy. Enzyme replacement therapy (ERT) with recombinant human lysosomal acid α-glucosidase has been trialled in humans with mixed results (Amalfitano et al. 2001; Klinge et al. 2005; Winkel et al. 2004). Studies in α-glucosidase KO mice suggest that excessive autophagic buildup, particularly in glycolytic fibres, underlies muscle damage (Fukuda et al. 2006). Moreover, this buildup has been hypothesized to prevent trafficking of replacement enzyme to the lysosomes and compromise ERT efficacy (Fukuda et al. 2006). This accumulation of autophagic material at the core of the fibres is due to impaired autophagosome lysosome fusion, although induction of autophagy may also contribute (Raben et al. 2008). Indeed, engineered MLCcre:Atg7^F/F^:GAA^−/−^ mice that combine α-glucosidase deficiency and skeletal muscle-specific impairment of autophagy show very significant reduction of autophagic buildup and good response to ERT compared to the lack of clearance of lysosomal glycogen observed in GAA^−/−^ mice (Raben et al. 2010). In humans, autophagy impairment manifested as accumulation of p62-positive aggregates correlates with atrophy both in infantile and late onset cases of Pompe disease (Nascimbeni et al. 2012). This study showed that autophagy acts as a protective mechanism during the early stages of the disease and may enable ERT efficacy; conversely, if excessive autophagic buildup and glycogen are already present, there is no beneficial response to ERT, possibly because autophagy is required for delivery of recombinant GAA to the lysosomes but is irreversibly compromised (Nascimbeni et al. 2012).

More recently, mutations have been identified in the ectopic p-granules autophagy protein 5 (EPG5) gene as causative of VICI syndrome (Cullup et al. 2013). EPG5 deficiency causes an autophagic block, with accumulation of numerous vacuole-like structures and dense bodies, possibly of lysosomal origin in skeletal muscle. Although the molecular function of EPG5 is not known, it appears crucial for the formation of degradative autolysosomes (Tian et al. 2010). This appears consistent with accumulation of p62, NBR1 and lipidated LC3-II in patient-derived fibroblasts, altogether pointing at an autolysosome clearance defect (Cullup et al. 2013).

A number of muscle diseases have also been reported in which alterations of autophagy flow contribute to the pathogenic mechanism. Autophagic signalling has been shown to be impaired in muscles from dystrophin-deficient mdx mice and Duchenne muscular dystrophy patients (De Palma et al. 2012; Pauly et al. 2012; Eghtesad et al. 2011). Reactivation of autophagy by dietary means, induction of AMPK activation or rapamycin treatment has been shown to be effective in ameliorating the dystrophic phenotype in mdx mice (De Palma et al. 2012). Delivery of rapamycin via direct intramuscular injections of nanoparticles is particularly effective in inducing autophagic flux both in wild type as well as mdx animals (Fig. 4 in Bibee et al. 2014). The latter study indicates that a role for mTOR-C1 (the rapamycin sensitive component of mTOR) in inducing autophagy exists in normal muscle, in contrast to previous findings showing that rapamycin treatment does not induce significant LC3 lipidation in skeletal muscle (Fig. S6 in Mammucari et al. 2007); the authors of this latter study unconventionally propose that mTOR-C2 rather than mTOR-C1 is essential for autophagy.

Mutations in the LMNA gene, which encodes lamin A and C (lamin A/C), cause autosomal Emery–Dreifuss muscular dystrophy. A mouse model carrying a point mutation in *Lmna* (*Lmna* ^*H222P/H222P*^) faithfully recapitulates the human disease. *Lmna* ^*H222P/H222*^ mice have enhanced mTORC1 signalling specifically in cardiac and skeletal muscle. *Lmna* ^*H222P/H222P*^ mice treated with the rapamycin analogue temsirolimus exhibit improved cardiac function with reduced expression of genes associated with ventricular dilatation relative to those treated with placebo (Choi et al. 2012). Similar results were obtained with another lamin-deficient mouse model of Emery–Dreifuss muscular dystrophy using rapamycin injections (Ramos et al. 2012). Thus, inhibition of mTORC1 led to significant amelioration of the cardiac pathology in both cases by efficient reactivation of autophagy with rapamycin or one of its analogues.

A number of mutations in mice and humans result in skeletal muscle pathology with changes in autophagy as a likely contributing factor. Loss of function mutations in myotubularins, the lipid phosphatases that specifically dephosphorylate PI3P and PI(3,5)P2 at the D3 position, has been associated with defects in the inhibition of autophagy and myopathy (Fetalvero et al. 2013). These include mutations in myotubularin 1 (MTM1) and myotubularin-related protein 14 (MTMR14) that are associated, respectively, with X-linked myotubular myopathy (Laporte et al. 1997) and congenital disease centronuclear myopathy (Tosch et al. 2006). Mutations in valosin-containing protein (VCP) cause inclusion body myopathy and Paget disease of the bone and have been shown to alter autophagosome maturation and autophagy impairment (Custer et al. 2010; Tresse et al. 2010). Additional examples with opposite effects on the levels of autophagy are provided by mutations in laminin a2 and collagen VI. Expression of autophagy-related genes is upregulated in laminin a2 chain-deficient muscle and, moreover, inhibition of autophagy significantly improves the dystrophic phenotype of the mouse model (Carmignac et al. 2011). Conversely, skeletal muscles of collagen VI–knockout mice show impaired autophagic flux, lower induction of beclin-1 and Bnip3 and a lack of autophagosomes after starvation. Forced activation of autophagy by genetic, dietary and pharmacological approaches restored myofibre survival and ameliorated the dystrophic phenotype of the knockout mice (Grumati et al. 2010). Expression of a mutant form of SOD1 (SOD1G93A) in mice has also been shown to cause elevated activation of autophagy due to increased oxidative stress, resulting in muscle atrophy (Dobrowolny et al. 2008), and sarcopenia also results from elevated autophagy (Wenz et al. 2009). Finally, ultrastructural evidence of autophagy and uncleared inclusions are present in a mouse model of hereditary inclusion myopathy (h-IBM), a distal myopathy caused by mutations in the UDP-*N*-acetylglucosamine 2-epimerase/*N*-acetylmannosamine kinase (GNE) gene, which encodes for a bifunctional enzyme involved in sialic acid biosynthesis (Malicdan et al. 2007). The underlying mechanisms of autophagy disruption in these and other examples (Chang et al. 2012; Bridges et al. 1992; Roos et al. 2014) (see also Table 2) remain largely unknown, but the panoply of mutations in mice and humans summarized here indicate that muscle is particularly vulnerable to the dysregulation of autophagy.

**Table 2:** Genes associated with skeletal muscle pathology with evidence of autophagy disruption

Gene	Disease/model	Evidence of autophagy disruption	References
VMA21	X-linked myopathy with excessive autophagy (XMEA)	Reduced lysosomal proton influx	Ramachandran et al. (2009)
LAMP2	Danon disease	Autophagosome accumulation	Tanaka et al. (2000), Nishino et al. (2000)
GAA	Pompe disease	Impaired autophagosome–lysosome fusion	Amalfitano et al. (2001)
EPG5	VICI syndrome	Autolysosome clearance defect	Cullup et al. (2013)
MDX	Duchenne muscular dystrophy	Impaired autophagy signalling	De Palma et al. (2012)
LMNA	Emery–Dreifuss muscular dystrophy	Enhanced mTORC1 signalling resulting in inhibited autophagy	Choi et al. (2012)
MTM1	X-linked tubular myopathy	Defects in autophagy inhibition	Fetalvero et al. (2013)
MTMR14	Congenital disease centronuclear myopathy	Defects in autophagy inhibition	Fetalvero et al. (2013), Tosch et al. (2006)
VCP	Inclusion body myopathy	Altered autophagosome maturation	Custer et al. (2010)
LAMA2	Mouse model	Constitutive upregulation of autophagy genes	Carmignac et al. (2011)
COL6A	Mouse model	Impaired autophagy induction and flux	Grumati et al. (2010)
GNE	Hereditary inclusion myopathy	Ultrastructural evidence of autophagy and uncleared inclusions	Malicdan et al. (2007)
KY	Hereditary kyphoscoliosis	Ultrastructural evidence of autophagy	Bridges et al. (1992)
SIL1	Marinesco–Sjogren syndrome	Impaired autophagic clearance	Roos et al. (2014)
SOD1	Mouse model	Elevated oxidative stress resulting in constitutively elevated autophagy	Dobrowolny et al. (2008)
DNAJB6	Limb girdle muscular dystrophy	Loss of autophagy co-chaperone	Sarparanta et al. (2012)
BAG3	Mouse model of fulminant myopathy	Central chaperone to Chaperone-Assisted Selective Autophagy	Arndt et al. (2010), Homma et al. (2006)
ATG7	Inducible deletion/muscle-specific deletion	Accumulation of dysfunctional mitochondria	Lira et al. (2013), Kim et al. (2013), Masiero et al. (2009)
BCL2	Mouse knock-in	Prevention of exercise-induced autophagy	He et al. (2012)
BECN1	Haploinsufficient mouse model	Impaired upregulation of autophagy	Lira et al. (2013)
CISD2 (NAF-1)	Mouse model of Wolfram syndrome 2	Enhanced basal autophagy	Chang et al. (2012)
VPS15	Autophagic vacuolar myopathy	Defects in late endosomal/lysosomal functions	Nemazanyy et al. (2013)

### Mechanical stress and proteostasis

It appears clear that autophagy is important for short- and long-term muscle adaptations to mechanical stress (He et al. 2012). Both the application and absence of mechanical stress in muscle result in a requirement to increase protein degradation. The application of mechanical stimuli results in elevated levels of unfolded protein, which must be cleared efficiently to prevent cell stress, toxicity and the formation of disruptive aggregates. As described below, prolonged paralysis evokes an adaptive increase in protein degradation that results in net disassembly of sarcomeric structures and whole muscle atrophy.

A key set of proteins in the activation of degradative pathways are the FOXO transcription factors (reviewed in Milan et al. 2015). These were identified as the primary contributor to upregulation of atrogene expression in skeletal muscle in atrophic conditions, as well as activation of autophagy pathways (Sandri et al. 2004; Mammucari et al. 2007). The FOXO transcription factors are suppressed by phosphorylation via AKT signalling (Stitt et al. 2004), and activated via HDAC1 in conditions which suppress AKT activity, such as limb suspension (Beharry et al. 2014).

Given the coordinate regulation of both degradative pathways, it would appear that both autophagy and UPS have a role in skeletal muscle adaptation during atrophy, though the relative importance of each pathway remains unclear. A study in a myoblast cell line showed that FOXO-induced autophagy substantially contributed to proteolysis in atrophy-inducing conditions (Zhao et al. 2007), though how well this translates in vivo has yet to be established.

The balance of evidence suggests that the UPS has a more critical role in adaptive atrophy, which is associated with elevated proteasome activity (Medina et al. 1991), upregulation of proteasome-encoding mRNA (Medina et al. 1995) and upregulation of the E3 ubiquitin ligases MuRF1, MAFbx (Bodine et al. 2001) and the recently identified SMART (Milan et al. 2015). Inhibition of the proteasome protects from starvation- (Caron et al. 2011) and denervation (Beehler et al. 2006)-induced atrophy. In contrast, inhibition of autophagy has been demonstrated to itself result in atrophy (Masiero et al. 2009; Masiero and Sandri 2010), but this is likely a secondary effect of disrupted muscle maintenance, with accumulation of damaged proteins and organelles impacting on tissue growth, a distinct mechanism from regulated atrophy. Given that inhibition of autophagy fails to protect from atrophy (Masiero et al. 2009), autophagy appears less likely to have a major role in adaptive atrophy.

Though there is some degree of upregulation of proteasomal subunit activity in mechanical overloading experiments (Baehr et al. 2014), the correlation between mechanical stress and autophagy is more established. Autophagy is elevated for around 24 h after exercise (Moller et al. 2015). Moreover, baseline levels of autophagy rise in adaptation to repeated bouts of exercise (Lira et al. 2013; Grumati et al. 2010; Ulbricht et al. 2015). Presumably, the former is a mechanism to aid clearance of damaged proteins and organelles immediately after mechanical stress, and the latter the mechanism required to couple higher protein turnover to continued protein unfolding.

The mechanistic link between mechanical stimuli and autophagy remains poorly defined, but there is strong evidence that the BAG (Bcl2-associated athanogene) proteins are involved in the adaptation of proteostasis. BAG proteins have multiple domains and are known to modulate a number of cellular processes (Kabbage and Dickman 2008) including the chaperone activity of heat shock protein Hsc70 (Brive et al. 2001). In the context of proteostasis, complexes containing BAG1 favour degradation via UPS, whereas complexes involving BAG3 favour degradation via autophagy (Behl 2011). This “molecular switch” from BAG1 to BAG3 signalling prominence has been proposed to mediate adaptive upregulation of autophagy during ageing and cell stress (Behl 2011; Minoia et al. 2014). It is likely that this mechanism is relevant to immediate repair and longer-term adaptation in muscle. Indeed, BAG3 expression is increased in tension, under the regulation of Heat Shock Factor 1 (HSF1) (Ulbricht et al. 2013) but whether the reciprocal expression of BAG1 and BAG3 seen in ageing exists in the skeletal muscle mechanotransduction context has yet to be explored. However, a recent study has demonstrated that BAG3 has a higher affinity than BAG1 for co-chaperones (Rauch and Gestwicki 2014), thus upregulation of BAG3 alone may fulfil the role of the molecular switch towards autophagy.

The question of why upregulation of autophagy, and not UPS, might be favoured in this context is likely one of efficiency. Whilst the relative energetic cost of autophagy and the UPS for equivalent clients remains unknown, evidence suggests that autophagy is better able to clear aggregates, as demonstrated in certain pathological models (Rodriguez-Navarro et al. 2010; Ruparelia et al. 2014; Schaeffer et al. 2012) and is certainly better able to turn over damaged or faulty organelles.

### The Z-disc integrates autophagic and hypertrophic pathways

Tension-induced growth signalling is by no means limited to proteins at the z-disc. For example, the kinase domain of titin has been proposed to induce elevated protein synthesis when activated by tension. The conformational change promotes activation of Neighbour of BRACA1 gene 1 (NBR1), which recruits p62 to the sarcomere. This facilitates MuRF2 activation of serum response factor (SRF), which is translocated to the nucleus to promote transcription (Lange et al. 2005). NBR1 also interacts with LC3-I and polyubiquitin chains, suggesting a link to protein turnover (Waters et al. 2009; Kirkin et al. 2009). As previously discussed, NBR1 puncta are observed in the muscles of VICI syndrome patients (Cullup et al. 2013). However, although a mutation in titin disrupting nbr1 interactions with the kinase domain of titin is associated with a muscular disorder (Lange et al. 2005), there are no specific models of NBR1 disruption with an overt muscle phenotype.

The Z-disc arose as the second most significantly enriched cellular component amongst muscle disease genes (Table 1). The Z-disc lies in series with the force-generating sarcomeres and experiences force directly, being therefore ideally placed to include triggers of hypertrophy upstream of the mTOR pathway, particularly in light of the increasing evidence that the mTOR pathway is distinctively activated by growth factors and mechanical stimulation (Miyazaki et al. 2011). Mutations underlying cardiac and/or myopathic disorders suggest that the Z-disc is also a mediator of muscle adaptation. For example, mutations in the Z-disc associated proteins T-cap, Myozenin-2, ZASP, myotilin, Filamin C, alpha–beta-crystallin, BAG3, FHL1, DNAJB6, alpha 2 actinin, desmin or *KY* provoke hypertrophy of the ventricular walls, dilated cardiomyopathy or skeletal muscle disorders (Blanco et al. 2001; Goldfarb et al. 1998; Hauser et al. 2000; Mohapatra et al. 2003; Moreira et al. 2000; Osio et al. 2007; Sarparanta et al. 2012; Schessl et al. 2008; Selcen et al. 2009; Vatta et al. 2003; Vicart et al. 1998; Vorgerd et al. 2005). These various pathologies reflect the functional impairment of the protein, but the fact that some mutations cause dysregulation of growth control in the heart has led to the current predominant view that signalling hubs for mechanosensation and mechanotransduction, amongst other locations such as the M-band or costameres (Gehmlich et al. 2008), must also reside at the Z-disc (Frank and Frey 2011; Frank et al. 2006). Despite the strong genetic evidence, the underlying mechanisms translating Z-disc based cytoskeletal stresses into gene expression remain poorly defined.

A tension-induced form of client-specific autophagy, CASA, has been recently described. CASA plays a major role in tension bearing cells and is required for muscle maintenance (Arndt et al. 2010; Ulbricht et al. 2013). The strain-provoked irreversible unfolding of the Z-disc and actin crosslinker protein filamin C (*FLNC)* is proposed to be the trigger of CASA. The chaperones HSC70 and HSPB8 bind to unfolded *FLNC* and form a complex with BAG3 that likely assists in releasing damaged *FLNC* from the Z-disc. *FLNC* is then ubiquitinated and complexed with autophagosome membrane precursors and degraded upon lysosomal fusion. Intriguingly, as discussed previously, mechanical tension also upregulates BAG3, making BAG3 available for interaction with components of the Hippo pathway. In particular, BAG3 interacts with inhibitors of the *YAP*/*TAZ* transcription factors via its WW domains, effectively releasing *YAP*/*TAZ* from its inhibitors. *YAP*/*TAZ* can then translocate to the nucleus and upregulate the synthesis of target genes involved in cytoskeleton remodelling (Morikawa et al. 2015), including *FLNC*. This dual role of BAG3 enables CASA to deal with mechanical stress whilst maintaining the correct balance of functional protein, by adapting turnover rates of *FLNC* to tension conditions (Ulbricht et al. 2013).

Although CASA components are upregulated in stressed muscle (Ulbricht et al. 2015), the CASA mechanism has been elucidated primarily in smooth muscle cells (Ulbricht et al. 2013). Extrapolation of CASA to sarcomeric cells is plausible, but the overall importance of CASA as sensor and mediator of cytoskeletal stresses in skeletal muscle is likely to rest on the identification of additional skeletal muscle-specific clients. The importance of CASA in muscle maintenance stems from Z-disc defects of BAG3 mutants in mammals and *Drosophila*. However, the Z-disc disorganization and other myopathic changes observed in the mouse BAG3 knockout (Homma et al. 2006) may at least be partially attributed to a structural role of BAG3 distinct from its co-chaperone function in CASA, as suggested by other evidence (Hishiya et al. 2010).

## Conclusions

Although key details remain to be addressed, autophagy is co-substantial to skeletal muscle maintenance and adaptation and has already been targeted to ameliorate disease (Raben et al. 2010; Carmignac et al. 2011; Grumati et al. 2010, 2011 Bhuiyan et al. 2013; Cabet et al. 2015; Chrisam et al. 2015; Hidvegi et al. 2015; Hsueh et al. 2016; Whitehead et al. 2015; Foltz et al. 2016). A greater understanding of tension-induced autophagy systems may help to elucidate connections between protein unfolding and mTOR-dependent or mTOR-independent hypertrophic responses. This is likely to reveal new and more specific therapeutic windows for the treatment of muscle wasting disorders.

## Electronic supplementary material

Below is the link to the electronic supplementary material.

Supplementary material 1 (XLSX 50 kb)

Supplementary material 2 (XLSX 63 kb)

Supplementary material 3 (XLSX 359 kb)
